# Education Research: Creating Online Interactive Case-Based Learning Experiences From Educational Case Reports With Large Language Models: A Feasibility Study

**DOI:** 10.1212/NE9.0000000000200250

**Published:** 2025-09-19

**Authors:** Christina Gao, Galina Gheihman, Tamara Kaplan, Liam G. McCoy, Luke C. Collins, Tara Wenzel, Ashley Paul, Haatem Reda, Laura Katherine Stein, Grace Kimbaris, Harry W. Sutherland, Sybil Stacpoole, Tracey A Milligan, Rudy Goh, Stephen Bacchi

**Affiliations:** 1 Department of Medicine, Adelaide Medical School, Adelaide, Australia;; 2 Department of Neurology, Mass General Brigham, Boston, MA;; 3 Department of Neurology, Harvard Medical School, Boston, MA;; 4 Department of Neurology, University of Alberta, Edmonton, Canada;; 5 Department of Neurology, Austin Health, Melbourne, Australia;; 6 Department of Neurology, Johns Hopkins University, Baltimore, MD;; 7 Department of Neurology, Icahn School of Medicine at Mount Sinai, New York, NY;; 8 Department of Neurology, University of Pennsylvania, Philadelphia, PA;; 9 Department of Neurology, Yale School of Medicine, New Haven, CT;; 10 Department of Neurology, University of Cambridge, Cambridge, UK; and; 11 Department of Neurology, New York Medical College, Westchester Medical Center, NY.

## Abstract

**Background and Objectives:**

Case reports are a fundamental part of medical literature and education. Artificial intelligence (AI) is increasingly influencing medical education and can potentially augment the delivery of the educational content in case reports. The aim of this study was to evaluate the feasibility of using AI, namely large language models (LLMs), to convert previously published *Neurology Resident & Fellow Section Case-based Articles (RFS-CBAs)* into an interactive online format to facilitate case-based learning.

**Methods:**

Three *RFS-CBAs* were converted into a free-text “screenplay” using the LLM Claude 3.5 Sonnet. These “screenplays” were then delivered in an interactive format through an online platform using GPT-4o. Two neurology fellows interrogated (prompted) the cases delivered by the online platform in a question-and-answer manner, seeking history, examination findings, and investigation results to arrive at a diagnosis and plan. These neurology fellows were not aware of the case report or screenplay content and asked questions in a manner that they would when evaluating a patient. A neurologist then reviewed each question-and-answer exchange for “screenplay” adherence and medical appropriateness. Results were analyzed with descriptive statistics.

**Results:**

The overall number of appropriate responses generated by the LLM was 206 of 210 (98.1%). There were 26 of 210 responses in which additional content was generated, all of which were medically plausible or consistent with the context of the case. The 4 errors that occurred were omissions of investigation results at the “screenplay” stage, which are amenable to manual correction. The omissions were the results of 3 unrevealing blood tests and 1 electroencephalogram result. None of these errors precluded the establishment of the diagnosis and completion of the case.

**Discussion:**

It is feasible to convert *RFS-CBAs* into an interactive question-and-answer format using LLMs. It should be noted that the nondeterministic nature of frontier LLMs and the potential for such LLM versions to change frequently are relevant considerations in making estimates of performance. Further studies investigating the impacts of this educational innovation are required.

## Introduction

Case reports are a fundamental part of medical literature and education, designed to share novel medical, scientific, or educational insights. Some case reports, such as the *Neurology Resident & Fellow Case-based articles* (*RFS-CBAs*), use a stepwise structure that may enhance learner engagement yet remain confined to static text on a page. Artificial intelligence (AI), in particular large language models (LLMs), offer novel ways to transform static case reports into interactive experiences, enhancing learning and engagement through immediate feedback that fosters deliberate practice.^1,2^

### AI and LLMs Are Influencing Medical Education

Advances in AI are reshaping how medical trainees learn, practice, and engage with educational content. Artificial intelligence broadly refers to the simulation of human intelligence by machines, encompassing abilities such as perception, reasoning, learning, and decision making.^3^ AI, namely LLMs, is rapidly transforming medical education by reshaping how learners seek information,^4^ prepare for assessment,^5^ take examinations,^6^ and engage in case-based learning.^7^ LLMs are deep learning models trained on massive text corpora to generate human-like language.^8^

The core strength of LLMs lies in their ability to interact in natural language. Unlike static resources, an LLM can engage in dialog, answer follow-up questions, and adapt its responses. Recent literature indicates that LLM-based generative AI can enrich learning by fostering more autonomous, collaborative, and interactive experiences.^9^ For example, a systematic review of 83 studies on LLM use in higher education highlighted that these models can personalize learning, provide immediate feedback, and promote student engagement.^9^

In medical education specifically, the past 5 years have seen *substantial interest* in harnessing LLMs as study aids, virtual tutors, and even simulators. A recent systematic review identified 40 relevant studies of LLM applications in medical training, with common themes including the promise of personalized learning and the pitfall of content inaccuracies.^10^ Indeed, LLMs are touted as a partial solution to information overload and faculty shortages by providing instant answers and explanations.^10^ These early use-cases illustrate how LLMs can function as simulated patients and virtual tutors—available at any hour to answer questions or provide practice cases—and thereby reinforce foundational knowledge in the preclinical curriculum. At the same time, students and educators have observed limitations. Although an LLM might excel at reviewing pharmacology facts or generating a list of diagnostic possibilities, it may falter in modeling the nuanced reasoning and ethical considerations that human mentors provide.^11^

Beyond examinations, neurologic education is exploring LLM-driven interactivity in clinical reasoning exercises. For example, a pilot study in 2025 examined LLM-supported case-based learning.^12^ There are many different types of case-based learning encounter. For example, another promising use of LLMs in neurology training is in simulated patient encounters. Neurology residencies often use standardized patients or role-play to teach history taking and communication, but these are resource-intensive. AI-driven virtual patients offer a new alternative. In 1 such study, a GPT-4 model was used to simulate a patient for medical students practicing clinical interviews, but not physical examinations or investigations.^13^

### Case Reports Are a Fundamental Component of Medical Education

Case reports, detailed narratives of unique or illustrative clinical cases, contribute meaningfully to the knowledge and training of health care professionals.^14^ Traditionally, case reports are presented in a static format, limiting opportunities for interactive learning. Although considered low on the evidence hierarchy, they can have outsized educational value. Each case report provides a contextualized account of a real patient encounter,^15^ bringing the abstract concepts of textbooks to life. Especially for trainees who lack extensive clinical experience, reading and discussing case reports serves as a proxy for “seeing” a wider variety of patients. Educational research supports that studying real-life cases improves knowledge retention and clinical reasoning skills by situating learning in a narrative.^15^ Case reports connect theory to practice—they highlight how the textbook presentation of a disease was modified by an individual's circumstances, how the clinicians' decisions were made in real time, and what the outcomes were.^15^ Case reports remain an integral pedagogical tool: they bring **real-world complexity** into medical training, build research and writing competency, and enrich the curriculum with lessons from unusual or instructive patient stories.^14^ Accordingly, the potential application of AI to these case reports holds significant potential.

### AI May Augment the Presentation of Case Reports, but Gaps in the Literature Remain

Although previous research in this area of using LLMs to deliver case-based content is promising, significant gaps in the literature remain. For example, previous studies have shown that LLMs can present information in a question-and-answer format when provided with tailored case descriptions, allowing learners to interact with a case as if taking a history, identifying examination findings, and requesting investigation results.^16^ In addition, related research suggests that LLMs can generate effective numerical scores based on learner performance.^17^ However, these previous studies were conducted with specifically constructed cases, which may differ from case reports in a number of manners, including length, structure, missing information, and used vocabulary. Whether previously published case reports, such as the *RFS-CBAs*, can be effectively adapted into an LLM-supported interactive format remains unclear. If this were possible, such open-access case reports could provide a significant resource for learners and increase the interactivity of this foundational element of medical education. The uncertainty as to the possibility of converting published case reports into an interactive format with LLMs reflects a gap in the literature.

Therefore, the aim of this study was to evaluate the feasibility of using LLMs to convert previously published *RFS-CBAs* into an accurate, interactive online format.

## Methods

### Overview

This study involved the conversion of 3 *RFS-CBAs*^18-20^ into “screenplays,” which were then presented through an LLM-based online platform to neurology fellows who interrogated (prompted) the cases. These interactions were then reviewed by a neurologist (eAppendix 1).

### Case Conversion Into “Screenplays”

**Figure 1:**
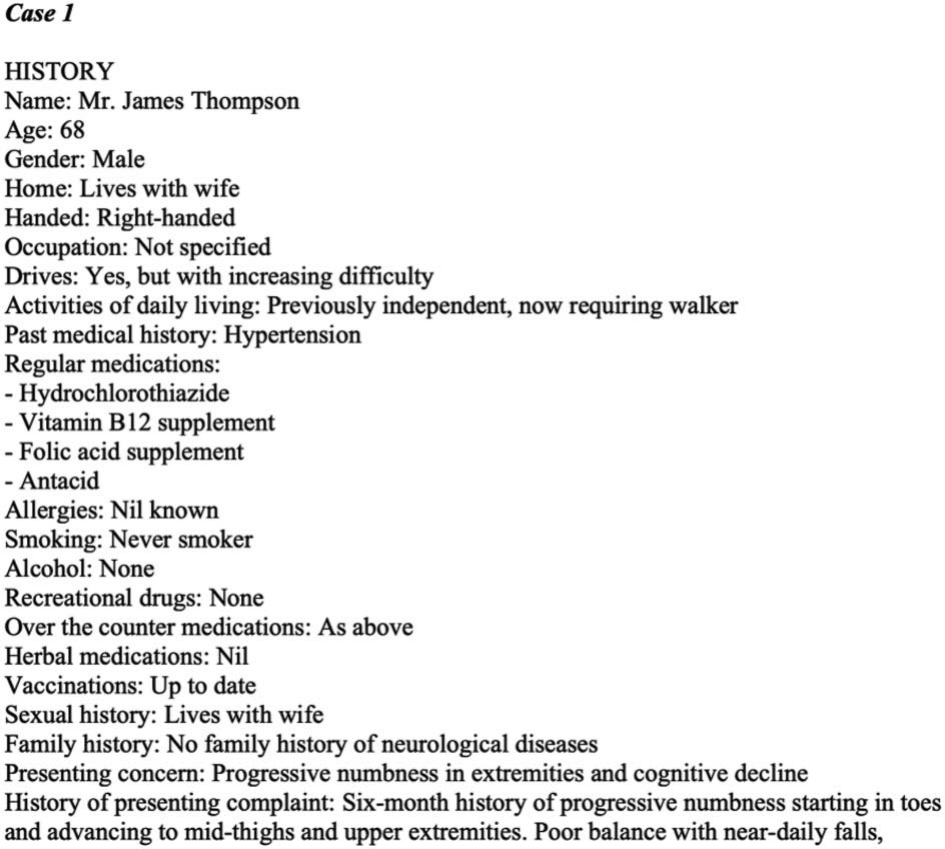
Example of the Beginning of a “Screenplay” Please also note that any additional details present in the following cases that were not present in the original case report (such as names) are fictional.

Three published clinical reasoning *RFS-CBAs* from 2024 to 2025 were selected randomly. These cases were then entered into *Anthropic*'s Claude 3.5 Sonnet, with a prompt (eAppendix 2) to convert them into a standardized dot point format (a “screenplay”) (Figure 1 and eAppendix 3). The prompt had been refined using content from another specialty and had not previously been applied to *RFS-CBAs* (eAppendix 2). For this study, no manual amendments were made to the screenplay before evaluation.

### Case Screenplay Presentation Through an Online Platform

**Figure 2:**
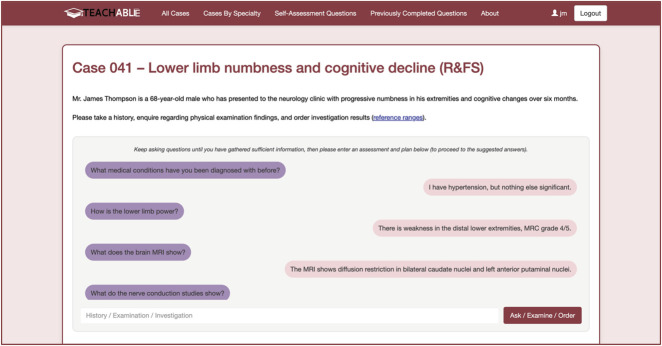
Demonstration of Interaction With the Online Platform

An investigator-developed LLM-based online platform was used to present the cases in an interactive format (TEACHABLE—Transforming Education And Clinical Healthcare through Agent-Based Learning and Evaluation, accessible online^21^). Through this platform, learners asked questions about the case, in the manner that they would ask history questions, seek physical examination findings, and request investigation results. This platform uses OpenAI's GPT-4o to answer learner questions based on the previously prepared “screenplay” (Figure 2).

### Case Presentation Evaluation

Because neurology fellows are the target audience for the RFS cases, 2 neurology fellows (T.W. and L.C.) individually interrogated (prompted, Figure 2) the 3 cases through the online platform, with the aim of arriving at a diagnosis and plan, with no limit on timing or the number of questions. These fellows were not aware of the content of the case reports or “screenplays” at the time of interrogating the cases. Investigator questions were entered into the online platform in a free-text manner, in English. The online platform generated screenplay-informed responses to each question. For example, each investigator taking the cases undertook 1 case in which Creutzfeldt-Jakob disease presented with peripheral neuropathy. In this case, history questions could include topics such as “How can I help you today?”, “When did the issue start?”, “What is your past medical history?”, and “What is your diet like?” In the physical examination section, questions were asked in a free-text manner again, with examples including “What is the plantar response bilaterally” and “How brisk are the angle jerks?” Similarly, in the investigation section, test results were requested in a free-text manner, such as “What are the vitamin B12 levels?” and “What does the MRI brain show?” The case concluded when the neurology fellows felt that they had discerned sufficient information to arrive at a final diagnosis and plan, which was subsequently recorded. This assessment and plan was entered by the investigators on completion of each of the cases.

All question-and-answer pairs were subsequently reviewed and encoded by a neurologist (S.B.). Each question-and-answer pair was evaluated separately (i.e., similar question types were not combined). The encoding was hierarchical, including whether the LLM

1. Provided an answer—determined whether the LLM attempted to answer the question instead of stating that it lacked sufficient information.
2. Declined to respond appropriately—evaluated whether the LLM's refusal to answer was justified based on the case content.
3. Provided an accurate answer—assessed whether the response was correctly based on the information presented in the case.
4. Generated new information—if the LLM produced an answer beyond the case details, judged whether the additional information was medically plausible.

### Data Analysis

Following the abovementioned algorithm, the proportion of responses that were appropriate was determined at each level of the hierarchy. The results were considered to support feasibility if the fellows were able to engage in questions and answers that were true to the original cases. No specific threshold for the proportion of appropriate responses required to demonstrate this feasibility was prespecified.

### Ethical Approval

This pilot study was performed using a publicly available LLM and involved only coinvestigators, and therefore, no ethics approval was required.

### Data Availability

Data will be made available, including individual AI answers, in response to reasonable request to the corresponding author.

## Results

### Case Characteristics

The 3 cases included diagnoses of Creutzfeldt-Jakob disease, spinocerebellar ataxia due to AFG3L2 heterozygosity, and neurolymphomatosis due to Sézary syndrome.^18-20^ The case investigations provided included blood test results, MRI findings, neurophysiology interpretations, lumbar puncture results, and histopathology reports. The screenplays that were generated for each of the cases had word counts of 449, 423, and 462, respectively (eAppendix 3).

### AI Answer Appropriateness

A total of 210 questions were posed to the LLM. For each of the fellows in case 1 there were 34 and 52 questions respectively. For case 2, there were 18 and 33 questions. For case 3, there were 21 and 52 questions, respectively.

The LLM provided answers to 182 of 210 questions (86.7%). In the 28 of 210 questions that the LLM declined to answer (e.g., “I do not have this information available”), it was correct to decline providing the information in 24 instances. There were 4 instances in which the LLM declined to provide information that was present in the original case. In all 4 instances, this occurred because the relevant information (investigation results—3 unrevealing blood tests and 1 EEG result, none of which were essential to establishing the diagnosis) had not been summarized in the step of converting the case into the “screenplay.” To correct this issue, the missing content has been added manually and highlighted in eAppendix 3.

Of the 182 LLM answers, 156 correctly presented content directly from the screenplay. There were 26 responses in which additional content was generated by the LLM. All such responses were considered medically plausible or appropriate within the context of the case (eAppendix 4).

Therefore, the overall proportion of appropriate responses was determined to be 206 of 210 (98.1%).

## Discussion

These findings demonstrate the feasibility of converting static, previously published *RFS-CBAs* into a dynamic, interactive format using LLMs. They also underscore the need for manual review of LLM-generated “screenplays” before use to ensure comprehensiveness, although only minimal edits are likely required. These findings support AI as a novel tool for delivering case-based educational content. It is possible that delivering peer-reviewed clinical cases as interactive learning experiences may allow learners to engage with clinically relevant content in a way that aligns with adult learning theory—specifically, through problem-solving rather than passive, content-centered instruction—although further research is required to demonstrate this possibility.^22^ These dynamic cases align with the Kolb Experiential Learning Cycle^23^ by fostering decision making (Concrete Experience), reflection through feedback (Reflective Observation), knowledge integration (Abstract Conceptualization), and application to future cases (Active Experimentation). For example, a learner may have to make a decision as to their assessment as to the cause of a peripheral neuropathy at the end of a case and then receive feedback on that case, before subsequently applying what they have learned to a future case.

The LLM-enabled system showed a high degree of accuracy. However, all systems, including LLMs, have a certain error margin. In this case, errors were mostly omissions rather than commissions (e.g., “hallucinations” by AI). In other words, there were no factually inaccurate pieces of information (e.g., “hallucinations”) identified in the question-and-answer pairs in this study, which is important to preserve the accuracy of the cases. A human-in-the-loop workflow could help to ameliorate these issues by checking screenplays for completeness and pilot testing cases. Furthermore, once deployed, such educational applications of LLMs would need to be monitored over time to ensure fidelity and to protect against model drift over time.

Looking ahead, the combination of LLMs with case-based teaching may promise significant possibilities. One exciting frontier is the development of sophisticated simulated patients and digital avatars for training. We have already seen GPT-4 credibly simulate a patient in text format, but future implementations could couple LLMs with voice and even visual avatars to create immersive virtual patient encounters. This allows students to interact in natural language and hone clinical skills in a safe environment. Digital patients can present with a range of symptoms and backgrounds, offering practice opportunities beyond the constraints of standardized patient actors.

These potential benefits must be weighed against the costs of LLMs. The use of proprietary frontier models is associated with a financial cost for the tokens used in both the creation of “screenplays” and generation of answers to user questions. An alternative approach may involve open-source LLMs. However, both proprietary and open-source LLMs may require significant amounts of energy, with associated environmental impacts.^24^

The potential for bias in LLMs is another consideration. For example, gender and racial biases have been observed in AI and LLMs.^25,26^ While the use of a previously written case may help to mitigate this issue, there is still potential for the issue to arise, particularly when factors such as race are not specified in a case report. Further research could examine this potential for bias through the conversion and evaluation of a larger number of case reports.

Another future direction is leveraging LLMs to increase exposure to rare diseases and scenarios that trainees might otherwise never encounter. As discussed, case reports have been a traditional mechanism to spread awareness of rare conditions. LLMs could support this learning by making rare disease case simulations readily accessible. If a student in a small training program in a low-resource setting wants to experience managing conditions such as neurosyphilis or neuromyelitis optica, they could potentially engage an LLM-driven simulator for that specific scenario. In fact, researchers have started to examine LLMs' proficiency with rare diseases. A recent study tested ChatGPT-3.5, GPT-4, and other models on complex diagnostic challenges involving rare diseases.^27^ All LLMs notably outperformed the average human participants in diagnosing these challenging cases. These findings suggest that AI could help to guide trainees through the reasoning for uncommon diagnoses. In the future, we might see AI-based tutoring systems specifically focused on rare neurologic disorders, ensuring that every trainee gets exposure to recognizing and managing conditions such as Wilson disease or inherited ataxias, regardless of patient volumes at their hospital. By democratizing expert knowledge, and through LLM case-based learning, LLMs can help bridge the gap between what is taught and the full breadth of what might be encountered in practice.

These innovations could improve access to quality medical education in low-income and underserved regions. Traditional simulation and extensive faculty-led teaching are often luxuries of well-resourced academic centers. The global reach of LLM-based education tools could help standardize and elevate medical training quality across disparate regions. For example, an aspiring neurologist in a low-income country could practice managing a virtual status epilepticus case guided by AI, gaining experience that local clinical opportunities might not afford. Of course, challenges remain in ensuring these tools are culturally and linguistically adapted, and that basic infrastructure (such as reliable internet) is in place. However, the vision of *AI-driven democratization of medical education* is coming into focus. Researchers assert that LLM-powered virtual patients will lay the foundation for worldwide, low-cost simulation training and will revolutionize how we teach clinical reasoning and decision making at scale.^28^

LLMs could perform well when delivering content and feedback on demand, personalizing the educational experience, and broadening exposure to rare knowledge. At the same time, they cannot (and should not) replace the human elements of medical training—mentorship, ethical judgment, and the tacit knowledge gained from real patient care. The pedagogical bedrock of case reports and case-based learning remains as important as ever, now complemented by AI that can generate and guide case discussions.

This pilot study used only *RFS-CBAs*, which are published in English. The model was tested with 2 learners who completed their training in Australia. Trainees from diverse centers may use different terminologies, which could affect LLM responses. Evaluation with diverse cohorts would be beneficial. It is also important to note the nondeterministic nature of frontier cloud-based LLMs such as those used in this study, and that repeated attempts may produce different results. Furthermore, frontier cloud-based models such as those used in this study may be updated over time as decided by the companies running these services, which could create future variation—this issue may be mitigated using open-source LLMs. While this study demonstrated the feasibility of converting published *RFS-CBAs* into an interactive LLM-supported model, further research is needed to evaluate its perceived utility among learners and its impact on educational goals such as clinical reasoning. Such future research should also seek to evaluate performance across learners at varying stages of training, from medical students to experts.

Using LLMs to convert *RFS-CBAs* into an accurate, interactive question-and-answer format is feasible. Errors were minimal and occurred during the generation of the “screenplay,” making them readily amenable to manual correction. Further study on the effectiveness of using LLM case delivery to support self-directed, active case-based learning is warranted, for both undergraduate and postgraduate learners.

## Conflict of Interest

The authors declare that there is no conflict of interest.

## Glossary

AI: artificial intelligence
LLM: large language model
*RFS-CBA*: *Resident & Fellow Section Case-based Article*

## References

[1] Preiksaitis C, Rose C. Opportunities, challenges, and future directions of generative artificial intelligence in medical education: scoping review. JMIR Med Educ. 2023;9:e48785. doi:10.2196/48785 37862079 PMC10625095

[2] McGaghie WC, Kristopaitis T. Deliberate practice and mastery learning: origins of expert medical performance. Researching Med Edu. 2022:315-324. doi:10.1002/9781119839446.ch28

[3] Xu Y, Liu X, Cao X, et al. Artificial intelligence: a powerful paradigm for scientific research. Innovation (Camb). 2021;2(4):100179. doi:10.1016/j.xinn.2021.100179 34877560 PMC8633405

[4] Richa Y, Asif A, Burton O, Richards H, Hennessey D. Changes in information seeking patterns of medical students in the social media and artificial intelligence age: preliminary findings. Clin Teach. 2024;21(6):e13772. doi:10.1111/tct.13772 38653544

[5] Law AKK, So J, Lui CT, et al. AI versus human-generated multiple-choice questions for medical education: a cohort study in a high-stakes examination. BMC Med Edu. 2025;25(1):208. doi:10.1186/s12909-025-06796-6 PMC11806894 39923067

[6] Mizori R, Sadiq M, Ahmad MT, et al. STEM exam performance: open- versus closed-book methods in the large language model era. Clin Teach. 2025;22(1):e13839. doi:10.1111/tct.13839 39496553 PMC11663729

[7] Stretton B, Kovoor J, Arnold M, Bacchi S. ChatGPT-Based learning: generative artificial intelligence in medical education. Med Sci Educ. 2024;34(1):215-217. doi:10.1007/s40670-023-01934-5 38510403 PMC10948641

[8] Clusmann J, Kolbinger FR, Muti HS, et al. The future landscape of large language models in medicine. Commun Med (Lond). 2023;3(1):141. doi:10.1038/s43856-023-00370-1 37816837 PMC10564921

[9] Peláez-Sánchez I, Velarde-Camaqui D, Glasserman-Morales L. The impact of large language models on higher education: exploring the connection between AI and education 4.0. Front Edu. 2024;9:1392091. doi:10.3389/feduc.2024.1392091

[10] Lucas HC, Upperman JS, Robinson JR. A systematic review of large language models and their implications in medical education. Med Educ. 2024;58(11):1276-1285. doi:10.1111/medu.15402 38639098

[11] Safranek CW, Sidamon-Eristoff AE, Gilson A, Chartash D. The role of large language models in medical education: applications and implications. JMIR Med Educ. 2023;9:e50945. doi:10.2196/50945 37578830 PMC10463084

[12] Gim H, Cook B, Le J, et al. Large language model-supported interactive case-based learning: a pilot study. Intern Med J. 2025;55(5):852-855. doi:10.1111/imj.70030 40125598

[13] Holderried F, Stegemann-Philipps C, Herrmann-Werner A, et al. A language model-powered simulated patient with automated feedback for history taking: prospective study. JMIR Med Educ. 2024;10:e59213. doi:10.2196/59213 39150749 PMC11364946

[14] Florek AG, Dellavalle RP. Case reports in medical education: a platform for training medical students, residents, and fellows in scientific writing and critical thinking. J Med Case Rep. 2016;10:86. doi:10.1186/s13256-016-0851-5 27048362 PMC4822269

[15] Jackson D, Cleary M, Hickman L. Case reports as a resource for teaching and learning. Clin Case Rep. 2014;2(5):163-164. doi:10.1002/ccr3.172 25614803 PMC4302617

[16] Gim H, Cook B, Le J, et al. Large language model-supported interactive case-based learning: a pilot study. Intern Med J. 2025;55(5):852-855. doi:10.1111/imj.70030 40125598

[17] Qian C, Gao C, Park S, et al. Medical Science Educator. Accepted Pending Online Publication; 2025.Use of large language models for rapid quantitative feedback in case-based learning: a pilot study. 10.1007/s40670-025-02343-6 PMC12228618 40625928

[18] Fanouraki S, Theodorou A, Velonakis G, et al. Clinical reasoning: progressive peripheral neuropathy in a 66-Year-Old woman with sezary syndrome. Neurology. 2024;103(10):e209983. doi:10.1212/WNL.0000000000209983 39442061

[19] Panigrahi B, Radhakrishnan DM, Saini A, et al. Clinical reasoning: a 50-Year-Old man with ataxia, dystonia, and abnormal ocular movements. Neurology. 2024;103(11):e210046. doi:10.1212/WNL.0000000000210046 39531603

[20] Regan SM, Davalos LF. Clinical reasoning: a 68-Year-Old man with progressive numbness, vertigo, and cognitive decline. Neurology. 2025;104(5):e213437. doi:10.1212/WNL.0000000000213437 39913879

[21] TEACHABLE: Transforming Education And Clinical Healthcare through Agent-Based Learning and Evaluation. 2025. Accessed January 2025. www.researchteaching.com

[22] Mukhalalati BA, Taylor A. Adult learning theories in context: a quick guide for healthcare professional educators. J Med Educ Curric Dev. 2019;6:2382120519840332. doi:10.1177/2382120519840332 31008257 PMC6458658

[23] Kolb D. Experiential Learning: Experience as the Source of Learning and Development. Prentice-Hall; 1984.

[24] Kleinig O, Sinhal S, Khurram R, et al. Environmental impact of large language models in medicine. Intern Med J. 2024;54(12):2083-2086. doi:10.1111/imj.16549 39542015

[25] Bacchi S, Teoh SC, Lam L, Schultz D, Casson RJ, Chan W. Bias, coronavirus, nationality, gender and neurology article citation count prediction with machine learning. Neurol Perspect. 2023;3(1):100115. doi:10.1016/j.neurop.2023.100115

[26] Menz BD, Kuderer NM, Chin-Yee B, et al. Gender representation of health care professionals in large language model-generated stories. JAMA Netw Open. 2024;7(9):e2434997. doi:10.1001/jamanetworkopen.2024.34997 39312237 PMC11420694

[27] Abdullahi T, Singh R, Eickhoff C. Learning to make rare and complex diagnoses with generative AI assistance: Qualitative Study of popular large language models. JMIR Med Educ. 2024;10:e51391. doi:10.2196/51391 38349725 PMC10900078

[28] Cook DA. Creating virtual patients using large language models: scalable, global, and low cost. Med Teach. 2025;47(1):40-42. doi:10.1080/0142159X.2024.2376879 38992981

